# Quality of Tuberculosis Care in Private Health Facilities of Addis Ababa, Ethiopia

**DOI:** 10.1155/2014/720432

**Published:** 2014-01-29

**Authors:** Gezahegn Gebrekidan, Gezahegn Tesfaye, Mitiku Teshome Hambisa, Negussie Deyessa

**Affiliations:** ^1^Policy and Planning Directorate, Federal Ministry of Health (FMOH), P.O. Box 50332, Addis Ababa, Ethiopia; ^2^Department of Public Health, College of Health and Medical Sciences, Haramaya University, P.O. Box 235, Harar, Ethiopia; ^3^School of Public Health, College of Medical Sciences, Addis Ababa University, P.O. Box 3253, Addis Ababa, Ethiopia

## Abstract

Ensuring provision of good quality tuberculosis (TB) care, especially in private for profit health facilities, is an important component of TB control strategy to reduce poor medical practice which results in multidrug resistant TB (MDR-TB). The aim of this study was to investigate quality of TB care in private health facilities of Addis Ababa. A facility based cross-sectional study was conducted based on Donabedian's structure-process-outcome model of health care quality. Quality of care was determined by adherence to National TB Program guidelines, treatment success rate, and client satisfaction. Exit interview was conducted on 292 patients on the intensive phase of treatment and 384 patient records were reviewed in eight private health facilities. Initial diagnostic AFB test was done for 95.4% of pulmonary TB patients. Most important components of TB care recommended by national guidelines were delivered for a significant proportion of patients. Majority (75%) of the clients were found to be satisfied with each component of TB care. The treatment success rate was 90.9%. The quality of TB care was fairly good. However, only 77.7% of the patients were counseled for HIV testing. Strengthening HIV counseling and testing, tackling shortage of streptomycin and laboratory reagent at private TB clinic is crucial.

## 1. Introduction

Tuberculosis (TB) remains a major global health problem. It causes ill-health among millions of people each year and ranks as the second leading cause of death from an infectious disease worldwide, after the human immune deficiency virus (HIV) [1]. The latest estimates indicate that there were 8.6 million new TB cases and 1.3 million TB deaths in 2012. Short-course regimens of first-line drugs can cure around 90% of cases, if the right treatment with recommended quality of care is provided [1].

DOTS (directly observed treatment, short course) is the internationally recommended control strategy for TB [2, 3]. This strategy includes the delivery of a standard short course of drugs to individuals diagnosed with TB. The delivery includes the direct observation of therapy (DOT), either by a health worker or by someone nominated by the health worker and the patient for this purpose (sometimes called a DOT supporter) [3].

The global target for TB control through full DOTS expansion was the attainment of 70% case detection and attainment of 85% cure rate by 2005 [4]. Though critical, these targets are insufficient in achieving the TB-related millennium development goals (MDGs) target of halting the spread and beginning to reverse the incidence of TB by 2015. Unfortunately, even these targets were not achieved, especially in Africa by the year 2005 and still only 82% [1, 5]. One major constraint identified as limiting the attainment of these targets is the noninvolvement of the private sector in the TB control programs. Thus, WHO observed that the target of 70% case detection would not be reached unless DOTS programs continue to expand geographically as well as involve the private sector consequently, and the current stop TB strategy that includes calls for promotion of public-private partnership was started in 2006 [5].

In 2011, the global TB treatment success rate was 87% among all new TB cases, while it was 89% in Ethiopia [1]. Ethiopia is one of the 22 high burden countries. According to the WHO global TB report 2011, there were an estimated 261 per 100,000 incident cases of TB in Ethiopia in 2010. According to the same report the prevalence of TB was estimated to be 394 per 100,000. During the year 2010/11, a total of 159,017 TB cases were notified in Ethiopia. Among these 151,866 (95.5%) were new cases of TB. The proportion of new smear-positive, smear negative, and EPTB among all new cases is 32.7%, 34.8%, and 32.5%, respectively. Only about 45% of TB patients have undergone HIV test and 15% were tested HIV positive [6].

To intensify the access and case detection rate, the Federal Ministry of Health (FMOH) has expanded DOTs services in line with WHO's global recommendation to involve the private sector in the delivery of TB services since 2006 which is known as public-private mix directly observed therapy-short course (PPM-DOTS) [6, 7].

Expanding TB care to the private sector increases access to care, particularly for clients who are reluctant to patient load at crowded public facilities and expand access to care for migrant populations who do not have local identity cards necessary to access care at public facilities [8].

Improving access to high-quality services also means reducing the harmful effects of poor medical practice. Inappropriate medical practices for TB diagnosis, treatment, and case management contribute to unnecessary suffering for patients, diagnostic delays, continuous spread of TB, high health-care costs for patients and society, and development of MDR-TB. Despite the increment of case detection rate through the engagement of private health facilities in TB care provision being encouraging, the emergence of drug resistance tuberculosis (MDR-TB) becomes a major public health problem in a number of countries including Ethiopia and an obstacle to the global TB control efforts [9].

Involvement of private sector for DOTS strategy in Ethiopia has been started since 2006 and this initiative increases access to service and case detection rate for TB control [8]. But to date, in Ethiopia, there is no study assessing the quality of provision of TB service in private health facilities. So assessing the quality TB care service is important to determine whether standards are being practiced in private health facility, to identify potential areas for improvement and to strengthen and implement better TB care in private health facilities.

## 2. Methods and Materials

### 2.1. Study Area

The study was conducted in Addis Ababa, the capital city of Ethiopia, which serves as the social, political, and economic center of the country. It is located at the geographic center of the country and covers a landmass of 540 km^2^ and has a total population of around 3 million. The city has 30 hospitals of which 25 are private, 29 health centers, 8 not-for-profit clinics, and 442 for-profit private clinics. In the city 25 private health facilities (10 hospitals and 15 higher clinics) provide DOTS service for more than two years during the study period [8].

### 2.2. Study Design and Period

**Figure 1:**
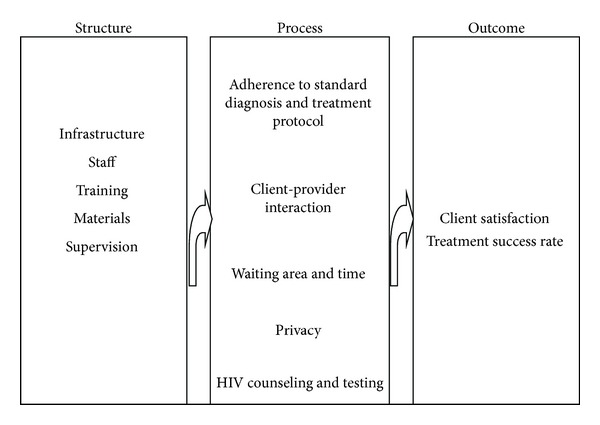
The conceptual framework for assessing quality of TB care in private health facilities of Addis Ababa, Ethiopia 2011.

A facility based cross-sectional study involving both quantitative and qualitative method was conducted. The study was based on the Donabedian's framework of health care quality assessment. Three dimensions of quality of TB care based on Donabedian's structure-process-outcome model were assessed [10]. The specific structural and process elements of TB care were identified from the National-TLCP manual and PPM-DOTS guidelines [6, 7] where availability of resources required to provide TB care and supervision are included for structural assessment, while technical performances, use of equipment and supplies for TB control activities, interpersonal relations, and convenience of TB care to patients are included for processes of care assessment. Patients' satisfaction level and treatment success rate were taken for outcome quality assessment (Figure 1). The study was conducted from March 11 to 22, 2011.

### 2.3. Sample Size and Sampling

**Figure 2:**
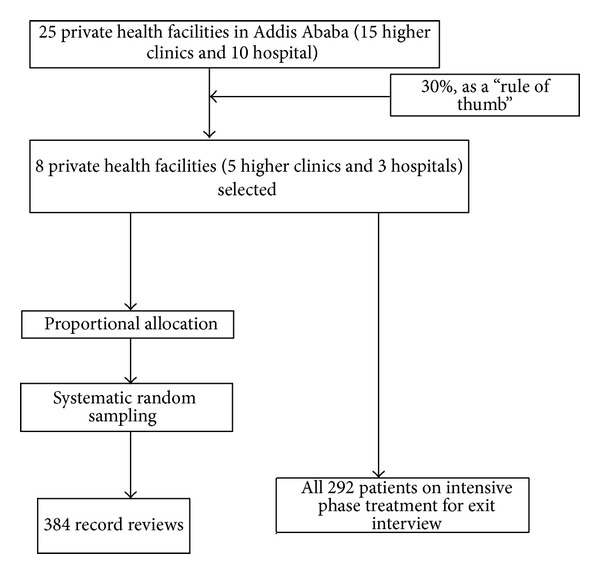
Schematic presentation of selection of health facilities and sampling strategies in private health facilities of A. A, 2011.

A “rule of thumb” was used for the rough estimation of sample size. According to this rule, for quality assessment of health care, if the numbers of units are less than 50, 30–50% of the sample will be taken [11]. Hence, eight private health facilities (five higher clinics and three hospitals), that is, 30% sample from a sampling frame of 15 higher clinic and 10 hospitals, were included in this study. Selection of eight private health facilities from sampling frame is done by simple random sampling (Figure 2).

With the purpose of assessing recent practices, patients who had completed their treatment in the previous one year in TB clinic were included for record review. The sample size for record review was determined by single population proportion formula based on the assumption that 50% of the patient record was complete, marginal error of 5% and CI 95% which yields a sample of 384. Health facility TB registration book was used for the sampling frame. A number of sampled TB patient who completed their treatment in the previous one year for each health facility were allocated proportionally based on the determined sample size and systematic random sampling technique was employed to select TB patient from TB registration book (Figure 2).

For exit interview, TB patients on intensive phase of treatment were included since they are available on daily basis for medication in the health facilities. Patients who visited the health facilities for the first time were excluded as they may not have adequate prior experience with the health facility to provide valid information. The total numbers of TB clients on intensive phase in the selected health facilities during the study period were 292. Hence, to get maximum sample size, all patients on the intensive phase of treatment in these eight private health facilities were included. In addition, observations on 71 patients were done to assess patient-provider interaction. TB control activities in the eight private health facilities were observed; heads and health workers in TB clinics were interviewed (Figure 2).

### 2.4. Measurements

The main outcome variables were patient satisfaction and treatment success rate, while independent variables include sociodemographic and socioeconomic variables such as age, sex, educational level, marital status, occupation, monthly income, availability and accessibility of services, adequacy of information, providers' competence in providing different services, initial diagnostic AFB test and result, HIV test and result, and classification of TB patient.

### 2.5. Data Collection

#### 2.5.1. Structural Assessment

National TB and leprosy control program (TLCP) performance monitoring checklist was used to assess availability of different materials, drugs, equipment, and supplies for TB control activities by the principal investigator. Data on staff assignment, training on tuberculosis control activities like AFB microscopy procedures, and patterns of service delivery and supervision were collected by interviewing heads of the health facilities.

#### 2.5.2. Process Quality Assessment

The principal investigator observed the process of care and reviewed record. Observations and health care providers' interview using guiding questions were made on some TB control activities like whether health education on TB is given in the health facility, time at which TB clinics opened, adequacy of information given to TB patients, patient's participation in decision-making process, and utilization of equipment in TB clinics and the level of provider-patient interaction. In addition to this, health workers in charge of TB clinic and laboratory technicians were also interviewed by the principal investigator on any procedures/norms followed in the health facility in the case detection, AFB microscopy procedures, treatment, monitoring and follow-up of TB patients, and use of guidelines, manuals, and so forth.

#### 2.5.3. Outcome Assessment

For the patient satisfaction level trained nurses conducted the exit interview using standard questionnaire among TB patients' on the intensive phase of treatment. Client's sociodemographic and socioeconomic characteristics, organization of treatment services, provider-client interaction, provider's competence, and adequacy of information were included in the interview. For the treatment success rate data from the record review was used.

### 2.6. Data Quality Assurance

Data collectors were trained for one day and the completeness, accuracy, and consistency of the collected data were checked on daily basis during data collection by the principal investigator. Incomplete, inaccurate and inconsistent questionnaires were returned back for data collectors to be filled again.

### 2.7. Data Processing and Analysis

Data were coded, cleaned, and entered into EPI info then transferred and analyzed using SPSS version 16 software for windows. Descriptive statistics were used to describe the structural, process, and outcome quality assessment results. Bivariate analysis was done by logistic regression to see any association between the outcome variables and independent variables. Since no variable was found to be significant on the bivariate analysis, further multivariate analysis was not done.

### 2.8. Ethical Considerations

Ethical clearance was obtained from University of Gondar institutional review board (IRB). Based on the ethical clearance, permission was obtained from A. A regional health bureau and the respective health institutions. Respondents were informed of the purposes, procedures, risks, and benefits of the study before making the interview. The privacy and confidentiality of the study participants were kept. Anonymity was maintained for all those records reviewed. For those patients less than 18 years old, oral consent was obtained from their parents and information has been collected from their parents.

## 3. Results

### 3.1. Sociodemographic Characteristics of Respondents

Exit interview of clients at TB service delivery outlet was carried out to assess their satisfaction level with the medical care for which the response rate of the study was 100%. All 292 clients on intensive phase of treatment were included in the study. More than half of respondents 52.4% were male, 63% were in the age group 1–35 years, 55.1% were grade 12 completed and above, majority 32.5% were private workers, 68.8% of them have income of less than 1500 Ethiopian birr, and more than half of them (51.7%) were in the first month of intensive phase (Table 1).

Regarding their means of transportation to get TB clinic, 56.8% got to the TB clinic by walking on foot and the remaining 43.2% clients used car/public bus to get TB clinic. The median time taken to reach the health facilities was 10 minutes, and the median waiting time to see their health care provider was 10 minutes, while the minimum and maximum waiting time was 1 and 40 minutes, respectively.

### 3.2. Resource Availability

TB care in all health facilities is provided in a separate room (Table 2). The TB rooms in each health facilities have light, ventilation, water supply, chair, table, and waiting space for TB clients. DOT service is opened throughout Monday to Friday from 8.30 am to 4.30 pm in all health facilities.

All the eight health facility has full time staffs assigned for TB clinic and TB care was run by trained TB nurses. Each health facility has at least one staff that had been trained on TB control activities and all of them had received refreshment trainings in the last 12 months (Table 2). All health facilities had at least one laboratory technician who had received AFB microscopy techniques where only four of health facilities had a laboratory technician who received refreshment trainings in the last 12 months.

Recent version of TLCP manual, TLCP laboratory manual, TB unit registration book, TB referral and transfer form, TB sputum examination request form, and TB control activity report form is available in all health facilities (Table 2). Only three health facilities have posted and used TB flip chart and flow chart for diagnosis of PTB+ and four health facilities had TB posters in different languages in a visible place. Except shortage of streptomycin, all of the health facilities had the recommended anti-TB drugs, namely, rifampicine, isoniazide pyrazinamide, and ethambutol, in the stock adequately during study period. As to the laboratory materials for TB diagnosis and control activities all of the health facilities provide routine laboratory tests, including HIV testing, microscopy for TB diagnosis (Table 2). All the required laboratory supplies based on the National TLCP implementation guideline are available in all health facilities except for a shortage of staining reagents due to inconsistent supply.

All health facilities, 8 (100%) had been supervised once in the last 6 months by Addis Ababa regional health bureau and other program supporters (Table 2). The supervision involved observation of TB registration book, discussion, and guidance in all health facilities; besides all supervised health facilities received written feedback timely.

### 3.3. Compliance with the National Guideline and Protocol

In depth interview with head of the health facility was done on service provision and all selected health facilities use the WHO recommended spot morning spot (SMS) sputum collection for AFB microscopic test. Besides, all the health facilities were using the recommended anti-TB drugs and their dosage based on NTLCP manual. Concerning drug provision to clients, on intensive phase, all eight health facilities provide the drugs to most of TB clients on daily basis under supervision, while some patient took the anti-TB drugs for 2–4 days for self-administration and came back after finishing. All health facilities monitored patients' treatment compliance by daily filling patient's TB registration form, pill count, and checking on monthly basis during continuation phase and all health facilities communicate contact person to trace absentee and defaulter. However, all health facilities had no health education program that addresses tuberculosis to their clients as part of their routine daily activities.

Record review was conducted on 384 patients who have completed their treatment in the previous one year. All of them were found to have a registered unique TB registration number. Out of 384 patients, 238 (61.8%) were pulmonary TB and 146 (38.2%) were extrapulmonary TB patients. Initial diagnostic AFB test was done for 59.1% of all TB patients (PTB and EPTB), where 37.4% were positive for AFB. From the pulmonary TB patients initial AFB test was done for 95.4%. During the continuation phase, weight was recorded for all patients, while drugs and their dosages given were recorded for 383 (99.7%) patients. Besides, follow-up AFB microscopy on the 5th/7th months of treatment were done for 78 (96.3%) of the 81 PTB+ patients, where one (1.23%) was found to be positive, 77 (95.1%) were negative, and it was unrecorded for one (3.7%) patient (Table 3).

Exit interview was done to assess anti-TB drug collection during intensive phase and HIV counseling and testing status. Of 292 TB clients, 227 (77.7%) were counseled and 65 (22.3%) were not counseled on HIV testing. Among TB clients counseled on HIV, 202 (89.0%) were tested, while the remaining 25 (11%) were not tested where the main reasons mentioned by clients were nonvoluntariness, laboratory cost, and tested before. Concerning anti TB drug collection, from the total of TB patient on intensive phase, 199 (68.2%) clients were collecting anti-TB drugs on daily basis under supervision from TB clinic, while the remaining 93 (31.8%) took the drugs home for self-administration and came back after finishing during the study period (Table 3).

Observation on 71 clients was done to assess provider-patient interactions while they receive the service in TB clinic. It was observed that all health workers in TB room of all health facilities demonstrate greeting, respectful, and encouraging attitude to their patients when they were receiving their drugs. Patients were seen in privacy in TB room and participated in part of decision making processes in the process of service delivery in each facility. Health information on the need to comply with treatment is provided in all health facilities.

### 3.4. Patient Satisfaction and Treatment Outcome

Clients' degree of satisfaction was assessed using different questions. It was found out that 10.3%, 7.5%, and 6.8% of study participants were dissatisfied in the adequacy and appropriateness of working hours, comfort of waiting area, and waiting time, respectively. Meanwhile, a higher proportion of study participants were satisfied with provider's competence/skill (99.3%), the measures taken to assure privacy (99.3%), and completeness of information given 98.3% (Table 4).

Scaling was done using the twelve satisfaction's related equations. The rating was determined using the count value with in cases' in the transform menu of SPSS software. Those TB clients who answered satisfied for each of the satisfaction related questions were taken as fully satisfied. Thus, the total clients who were satisfied fully in their stay at the day of their TB care visit were 219 (75%).

With respect to treatment outcome, out of all 384 patients, 79 (20.6%) were cured cases and 270 (70.3%) completed treatment, defaulter constituted 1 (0.3%), and the treatment success rate (those cured + treatment completed) was 349 (90.9%).

On the bivariate analysis there was no significant association between patients' satisfaction level and different sociodemographic characteristics of the patients. Again, there was no significant association between treatment success rate and the independent variables.

## 4. Discussion

This study showed that a significant proportion of patients attending TB clinic in the private health facilities have got important components of TB care recommended by the national guidelines. All of the health facilities had the recommended anti-TB drugs except shortage of streptomycin. Quantity and the qualities of staffing were satisfactory. Initial diagnostic AFB test was done for 59.1% of patients. Follow-up AFB microscopy on the 5th/7th months of treatment was done for 96.3% of the PTB+ patients, where only one was found to be positive. Majorities (75%) of the clients were found to be satisfied with each component of TB care they received and the treatment success rate was 90.9%.

Even though both the quantity and the qualities of staffing were satisfactory, only half of the health facilities have a laboratory technician who received refreshment training on TB control activities which is consistent with a study in Afar region where both the quantity and the qualities of staffing were not satisfactory in that almost half of the health facilities lacked laboratory technicians who received on the job training on TB control activities [12]. Staff development in general is a critical component of any DOTS program. The challenges of maintaining a competent and sufficient workforce remain problematic in TB high-burden countries [13]. This problem is confounded by high staff turnover. Like the public sector, the private sector is observed to be in great need of in-service training activities to update providers on new activities such as the Stop TB Strategy, including the management of TB linked to HIV/AIDS, multidrug resistant TB, and PPM-DOTS.

The supervision pattern seems good in all health facilities where all got a chance of being supervised in the last six months. The supervision pattern was also scheduled, consistent, and involves observation of TB registration book, discussion, and guidance in all health facilities and written feedback is given. This is consistent with the national recommendations where they recommend strong supportive supervision as a way of ensuring staff competence, effectiveness, efficiency, and satisfaction through observation, discussion, record reviewing, support, and guidance [6] as opposed to the study in Afar where no TLCP focal person had regular supervisor and supervisory schedule for TB control activities which may be due to the fact that there is usual strict follow up of quality service provision in the private health sector than the government sector [12].

All health facilities seem to be well equipped with the materials required for TB control activity as per the national standard. However, shortages of streptomycin drug and laboratory reagents were reported by providers despite the national TLCP guideline recommend adequate and consistent supply of TB drugs and other consumables [6]. This is in line with an evaluation assessment in Addis Ababa and Oromia which showed that the supply of the combination drug RH, INH, and E was not sufficient to last for more than three months and vitamin B6 was not available in all the facilities assessed and the available RHZE, E, and Streptomycin had a shelf life of less than three months [14]. In the same manner, in a study conducted in government health facilities in Jimma Zone, South West Ethiopia, shortage of laboratory reagents and slides for sputum smear microscopy were problems identified [15]. It is obvious that drug supply management and supply chain management are deficient and have the potential to hamper the delivery of quality TB services in the private sector [14]. However, it must also be noted that drug supply management is an inherent problem in the health care system in general and, as such, is faced by public health facilities as well.

The accuracy and completeness of the patient record may result in either underestimating or overestimating some of the indicators. The correct completion of patients' registration book is crucial to the patients monitoring and evaluation. This study revealed that the majority, 373 (97.1%) patient records', were found complete; that is, treatment for most patients was initiated and continued with proper recording of full information which is critical for patient monitoring. This finding was almost similar to study in Tigray where 93.2% of patients' records were found to be complete but higher than study conducted in Afar (11.5%) [12, 16]. The difference might be explained in terms of geographical, health infrastructure, and staffing difference between these areas as Addis Ababa is the capital of the country where there is high health infrastructure and highly qualified health professionals.

Sputum microscopy is the main diagnostic tool for pulmonary tuberculosis (PTB). All suspected TB cases should have sputum microscopy as their first diagnostic tool. But in our case, initial diagnostic AFB test was done only for 59.1% of patients. This finding is slightly lower than study conducted in Jimma in which 1st smear sputum microscopy was done for all 399 (100.0%) pulmonary TB patients [15]. This could be attributed to the fact that in our study there is the lack of laboratory reagent and lack of refresher training for laboratory technicians. Followup on the 2nd months of treatment seems good (95.3%) in that almost all initially diagnosed PTB+ cases received follow-up AFB microscopy which is in line with the national guideline, where it recommends that all PTB+ patients should get follow-up AFB microscopy services. Similarly, follow-up AFB microscopy at the 5th/7th months of treatment was done for 78 (96.3%) of diagnosed PTB+ patients, which is again in line with the FMOH's recommendations [6].

Information Education Communication (IEC) activities for TB control activities were found to be poor as even no one health facility was giving health education that addresses TB. Only half of health facilities have TB posters in different languages being posted in visible public places, despite its cost effectiveness. Similarly, a study in Jimma revealed absence of health education for TB patients, flip charts, and TB posters in local language were major identified problems [15]. Moreover, the research conducted in Afar showed that IEC activities for TB control activities were absent as only small proportion of health facilities were giving health education that address TB and had TB posters in different languages being posted in visible public places [12]. This again opposes the national guideline where every treatment facility is expected to deliver health education to patients and the public [6] as this is found to be the most effective and efficient strategy in health care programs implementation and interventions. The findings in the above studies show that IEC activities are ignored and less utilized in both private and public health facilities.

The patterns of patient-provider interactions were good in that almost all TB patients were greeted politely, participated in parts of decision making, advised to comply with treatment, and speak the same language with the providers. This seems that the processes of care were patient centered services which are in favor to the principles of quality health care and continuous quality improvement approaches [17]. Adequate communications between providers and patients are expected phenomenon in private health facility setup unlike the public health facilities and can be taken as an advantage to potentiate TB control activities.

Getting patients regularly to collect their drugs daily under supervision during intensive phase is recommended by the national TLCP manual. However, it is reported by providers and TB clients that a relatively high number of TB patients took drugs for 2 to 3 days home for self-administration and come back after finishing which affects TB control activities. This may be due to poor physical access to health facility, transport cost by clients, work load by providers, trust between clients and providers, and time inconvenience by client. Supporting bodies of evidence have reported from a study conducted in Gambia in which high defaulter rate was found among those patients that incurred significant time traveling to receive treatment [18].

Monitoring treatment adherence of TB clients is strongly recommended by WHO and national TB guideline [6] for implementing effective treatment compliance; the findings of this study showed that monitoring for treatment compliance was practiced in all health facilities through pill count, ensuring followup and providing accurate information, which could be again due to having relatively high number of trained health care providers and having strong regular supportive supervision from the respective bodies. This was supported by the study in Ethiopia which shows that patients tended to interrupt and default from treatment when their care provider had been inadequately supervised by district TB control experts [19].

The synergy between TB and HIV/AIDS is strong. In high HIV prevalence populations, TB is a leading cause of morbidity and mortality, and HIV is fuelling the tuberculosis epidemic in Ethiopia [20]. World Health Organization recommended package of collaborative TB/HIV activities to reduce the burden of TB/HIV includes HIV testing for TB patients [21]. However, record review of this study revealed that only 59.9% TB clients who had completed treatment in the previous one year got HIV test. On the other hand, from exit interview, 202 (69%) were tested. This finding is relatively high as compared to the national (45%) [6]. This high achievement as compared to the national may be due to free availability of HIV test kits to private health facilities and the trainee's motivation heightens through training of health worker on TB/HIV service. But still the target to reach the recommended 80% HIV testing rate among TB patients in Ethiopia is not yet achieved [22].

Satisfied client is more likely to comply with prescribed medical treatment and completion of treatment which is of utmost priority for TB control programs. Client satisfaction with the services and perceived quality tend to influence utilization of service as well as compliance with practitioner recommendation [23]. In this study the majority (75%) of the respondents were satisfied with all components of TB care that they received. But study conducted in Sidama zone, South Ethiopia, revealed that 90% of the study participants were satisfied with TB treatment service. [24]. This might be due to the difference in study setting and measuring clients' satisfaction might overestimate the satisfaction level in Sidama study, since the patients may respond in a relatively positive way fearing being recognized and similarly mostly satisfied patients usually visit public health facility. However, relatively higher proportions of TB clients were dissatisfied in the adequacy and appropriateness of working hours 133 (63.6%). Similarly most studies showed that waiting time or time spent with the provider strongly influence the level of client satisfaction [25–27], which can lead to service rejections by the patients and defaulting which can lead to incomplete treatment, treatment failure, and drug resistances.

The treatment success rate in this study was 90.9% which is similar with the national figure (89%) and a bit higher than the treatment success rate in Africa in 2011. This finding is also above the international target which is 85% [1]. This higher treatment success rate might be due to the good quality and quantity of staffs in private health facility, rational diagnosis, standard treatment, and successful followup (adherence to the national guideline).

The strength of this study was that it involves different approaches of data collection such as exit interview, record review, and observation, based on the Donabedian framework of health care quality assessment and focused on private for profit health facilities. Nevertheless, the study has the following limitations: reviewed records lack important variables and the study includes only private health facilities in A. A, so it may not represent all private facilities in Ethiopia.

## 5. Conclusion 

All health facilities have adequate resources to provide TB care. However, there is shortage of streptomycin TB drugs and inconsistent supply of laboratory reagent for AFB in all facilities. Adherence to national TLCP guidelines was high in all private health facilities as all health facilities were used SMS sputum collection for AFB test, monitor clients' treatment adherence, follow-up AFB test at 2nd and 5/7th for PTB+, and maintaining a standardized recording and reporting TB activities which are the most important aspect of DOTS to prevent and control TB and the development of MDR-TB. IEC activity on TB control, HIV counseling, and testing in TB clinic was poor. Majority of TB clients were found satisfied with each component of TB care. The treatment success rate was very good. Strengthening TB/HIV collaboration activity through offering HIV counseling and testing actively and routinely to all TB patients, tackling shortage of streptomycin and laboratory reagent in private TB clinics is crucial.

## Figures and Tables

**Table 1:** Sociodemographic characteristics of TB patients in private health facilities of A. A, 2011.

Variables	Number (*n* = 292)	Percent
Age group		
≤35	184	63.0
35^+^	108	37.0
Sex		
Male	153	52.4
Female	139	47.6
Marital status		
Single	131	44.9
Married	116	39.7
Divorced	32	11.0
Widowed	13	4.5
Educational status		
Illiterate	16	5.5
Elementary	48	16.4
Secondary	67	22.9
Grade 12 completed	98	33.6
Higher education	63	21.6
Occupation		
Government employee	95	32.5
Private worker	83	28.4
House wife	19	6.5
Merchant	49	19.5
Student	33	11.3
Others	13	4.5
Treatment duration		
On 1st month	151	51.7
On 2nd month	141	48.3
Income		
1–1500 Ethiopian birr	201	68.8
>1500 Ethiopian birr	91	31.2

**Table 2:** Summary of selected structure indicators in private health facilities of A. A, 2011.

Variables	Number (facility)	Percent
Separate TB room		
Yes	8	100
No	0	0
Presence of trained TB care provider		
Yes	8	100
No	0	0
Availability of standard monitoring tools		
Yes	8	100
No	0	0
Posted TB poster in different languages		
Yes	4	50.0
No	4	50.0
Recommended anti-TB drugs		
Rifampicine	8	100
Isoniazide	8	100
Pyrazinamide	8	100
Ethambutol	8	100
Streptomycine	0	0
Presence of HIV and AFB test		
Yes	8	100
No	0	0
Supervisory support in the last 6 months		
Yes	8	100
No	0	0

**Table 3:** Summary of selected process indicators in private health facilities of A. A, 2011.

Variables	Number	Percent
Record review (384)		
Initial AFB test done		
Yes	227	59.1
No	157	40.9
HIV test done		
Yes	230	59.9
No	154	40.1
Follow-up AFB microscopy done on 2nd month of Rx (85)		
Yes	84	95.3
No	1	4.7
Follow-up AFB microscopy done on 5/7th month of Rx (81)		
Yes	78	96.3
No	16	3.7
Completeness of information on TB registration		
Complete	373	97.1
Incomplete	11	2.9

Client exit interview (292)		
HIV counseling done		
Yes	227	77.7
No	65	22.3
HIV testing done (227)		
Yes	202	89.0
No	25	11
Health education program on TB		
Yes	0	0
No	8	100
DOT (collecting anti-TB drugs on daily basis under supervision)		
Yes	199	68.2
No	93	31.8

**Table 4:** TB patients' satisfaction level with different components of services in private health facilities of A. A, 2011.

Aspects of the variable	Satisfied	Neutral	Dissatisfied
Adequacy and appropriateness of working hours	262 (89.7%)	0 (0.0%)	30 (10.3%)
Waiting time	271 (92.8%)	1 (0.3%)	20 (6.8%)
Time spent with HW	281 (96.2%)	3 (1.0%)	8 (2.7%)
Cleanliness of waiting area	276 (94.5%)	4 (1.4%)	12 (4.1%)
Comfort of waiting area	265 (90.8%)	5 (1.7%)	22 (7.5%)
Cleanliness of examination/treatment room	269 (92.1%)	7 (2.4%)	16 (5.5%)
Cleanliness of treatment/diagnosis equipment	268 (91.8%)	16 (5.5%)	8 (2.7%)
Respect offered by health provider	287 (98.3%)	3 (1.0% )	2 (0.7%)
Measures taken to assure privacy	289 (99.0%)	2 (0.7%)	1 (0.3%)
Provider's competence/skill	290 (99.3%)	1 (0.3%)	1 (0.3%)
Cost incurred	286 (97.9%)	0 (0.0%)	6 (2.1%)
Completeness of information given	288 (98.3%)	2 (0.7%)	2 (0.7%)
